# CT-Based Pericardial Composition Change as an Imaging Biomarker for Radiation-Induced Cardiotoxicity

**DOI:** 10.3390/cancers17162635

**Published:** 2025-08-13

**Authors:** Arezoo Modiri, Ivan R. Vogelius, Cynthia Terrones Campos, Denis Kutnar, Jean Jeudy, Mette Pohl, Timm-Michael L. Dickfeld, Soren M. Bentzen, Amit Sawant, Jens Petersen

**Affiliations:** 1 Department of Radiation Oncology, School of Medicine, University of Maryland, Baltimore, MD 21201, USA; sbentzen@som.umaryland.edu (S.M.B.); asawant@som.umaryland.edu (A.S.); 2 Department of Oncology, Rigshospitalet, 2100 Copenhagen, Denmark; ivan.richter.vogelius@regionh.dk (I.R.V.); cynthia.terrones.campos@regionh.dk (C.T.C.); denis.kutnar@regionh.dk (D.K.); mette.poehl@regionh.dk (M.P.); jens.petersen@regionh.dk (J.P.); 3 Faculty of Health and Medical Sciences, University of Copenhagen, 2200 Copenhagen, Denmark; 4 Department of Computer Science, University of Copenhagen, 2100 Copenhagen, Denmark; 5 Diagnostic Radiology, School of Medicine, University of Maryland, Baltimore, MD 21201, USA; jjeudy@som.umaryland.edu; 6 Division of Cardiovascular Medicine, University of Maryland Medical Center, Baltimore; MD 21201, USA; tdickfel@som.umaryland.edu

**Keywords:** adverse outcomes, computed tomography, imaging biomarkers, lung cancer, pericardium, radiation-induced cardiotoxicity, radiation therapy

## Abstract

Background/Objectives: No reliable noninvasive biomarkers are available to predict RT-induced cardiotoxicity. Because the pericardial sac is a fast responder to cardiac injury, we investigated whether RT-induced radiographic pericardial changes might serve as early imaging biomarkers for late cardiotoxicity. Methods: We performed a retrospective study of 476 patients (210 males, 266 females; median age, 69 years; median follow-up, 26.7 months) treated with chemo-RT for small cell and non-small cell lung cancers at one single institution from 2009 to 2020. The heart and its 4 mm outmost layer (representing the pericardial sac) were contoured on standard-of-care baseline CTs. Six-month post-RT follow-up CTs were deformably registered on the baseline CTs. Data were harmonized for the effect of contrast. We labeled voxels as Fat, Fluid, Heme, Fibrous, and Calcification using Hounsfield units (HUs). We studied pericardial HU-change histograms as well as volume change and voxel-based mass change in each tissue composition. Results: Pericardial HU-change histograms had skewed distributions with a mean that was significantly correlated with mean pericardial dose. Voxels within Fluid, Heme, and Fibrous had mass changes consistent with the dose. In Kaplan–Meier curves, Fibrous and Heme volume changes (translating into thickening and effusion), Fat mass change, mean doses to heart and pericardium, history of cardiac disease, and being male were significantly associated with shorter survival, whereas thickening and effusion were significantly associated with shorter time to a post-RT cardiovascular disease diagnosis. Conclusions: Pericardium composition distribution has dose-dependent changes detectable on standard-of-care CTs at around 6 months post-RT and may serve as surrogate markers for clinically relevant cardiotoxicity. The findings should be validated with additional research.

## Simple Summary

Radiotherapy (RT) plays a vital role in eradicating tumors in many lung cancer patients. However, because of proximity to the heart, radiation adversely affects cardiac health. Finding a marker to identify patients who will need cardiac care has been the focus of investigation since RT-induced cardiotoxicity was clinically recognized. The pericardial sac, surrounding the heart, responds to damage to itself and to other cardiac parts. We hypothesize that pericardial health after RT could serve as a marker for identifying patients in need of cardiac care.

## 1. Introduction

As a primary modality for cancer treatment, radiation therapy (RT) can induce comorbidities by damaging healthy tissue. In particular, radiation-induced cardiotoxicity often joins the primary disease (cancer) to form a mutually reinforcing cycle that leads to poor outcomes [1,2,3,4,5]. Logotheti et al. summarized the variety of RT-induced cardiovascular diseases (CVDs) in their review article [6]. Studies showed that cancer survivors who received RT had a 1.7- to 2-fold increase in risk of cardiovascular death [2]. After Darby et al.’s seminal study suggested no safe dose threshold to avoid RT-induced cardiotoxicity [7], there has been an increased effort to manage cardiotoxicity, along with a search for proper cardiological evaluation for RT patients [8,9,10]. However, the latter task remains in the investigation stage, especially in lung cancer patients because of the close connection between cardiac and pulmonary systems. A retrospective study of 77,149 cancer patients showed that at the time of diagnosis (2014 to 2022), 59.7% of patients had at least one comorbidity, with the highest prevalence in lung cancer, with CVD being the most frequent comorbidity [11]. Adding to the complexity, research findings on this topic are not in agreement. For example, one study found a significant association between overall survival of lung cancer patients and dose to the left anterior descending artery [12], whereas another did not [13].

Outside the field of RT, studies have shown that pericardial sac abnormalities are early predictors of cardiac death [14,15,16,17,18,19]. Within the field of RT, studies have shown correlations between dose to pericardium and mortality risk [20,21]. Historically, the most prevalent and earliest RT-induced cardiotoxicity signs are radiation-induced pericardial changes [22,23]. However, the pathophysiological stages of RT-induced pericardial diseases are not well understood. Probably because the pericardium is an encompassing structure, the risk of pericardial disease has been found to be correlated with dose to various cardiac structures, including whole heart, right and left atrium, and the pericardium itself [4]. Once pericardial toxicity becomes clinically relevant (or symptomatic), it is usually associated with an unfavorable prognosis [16]. Pericardial diseases have gained new clinical interest and are being revisited in several recent studies [24,25,26]. On a CT, the normal pericardium is seen as a thin (≤2 mm) line of fibrous tissue, a small amount of fluid (between 15 and 50 mL), and some fat. While differences between normal and variant pericardial anatomy can be subtle, they could be important early markers for later CVD [18,27]. Although assessing pericardial health has been recommended as a part of clinical practice for identifying patients at greater risk of developing CVD [28,29], to our knowledge, no widely or routinely used clinical protocol has been reported, especially for cancer patients.

Here, we investigate pericardial composition changes detectable on standard-of-care images and their possible correlation with patient survival and late cardiac events. The scope of this study is limited to pericardium sac assessment based on CT images at the second follow-up (~6 months) after RT for lung cancer patients receiving chemo-RT.

## 2. Materials and Methods

### 2.1. Patient Data

Our original patient number was 1751, from which were excluded those with missing data (6-month follow-up CT scans with whole heart captured in both baseline and follow-up CTs) and patients with RT for multiple cancers within the studied period of time. All patients were treated for lung cancer (small cell (SCLC) or non-small cell (NSCLC)) in the Rigshospitalet from 2009 to 2020. Of the remaining 476 patients in our study, 183 had hypofractionation, and 293 received normal fractionation. NSCLC patients with curative intent received 66 Gy in 33 fractions (fx). Stereotactic body radiation (SBRT) doses for localized tumors <5 cm were 67 Gy in 3 fx or 50 Gy in 5 fx. If ultracentral, doses were 56 Gy in 8 fx. In SCLC, most patients received doses of 1.5 Gy/fx in 30 fx. An equivalent dose in 2 Gy fractions (EQD2) was used for our analysis and throughout this report. The 183 SBRT patients in our studied cohort received much lower heart doses (mean heart dose, ≤12.3 Gy; median, 0.3 Gy) than the 293 normally fractionated patients (mean heart dose, ≤32.1 Gy; median, 3.2 Gy). See Table 1 for patient characteristics.

**Figure A1:**
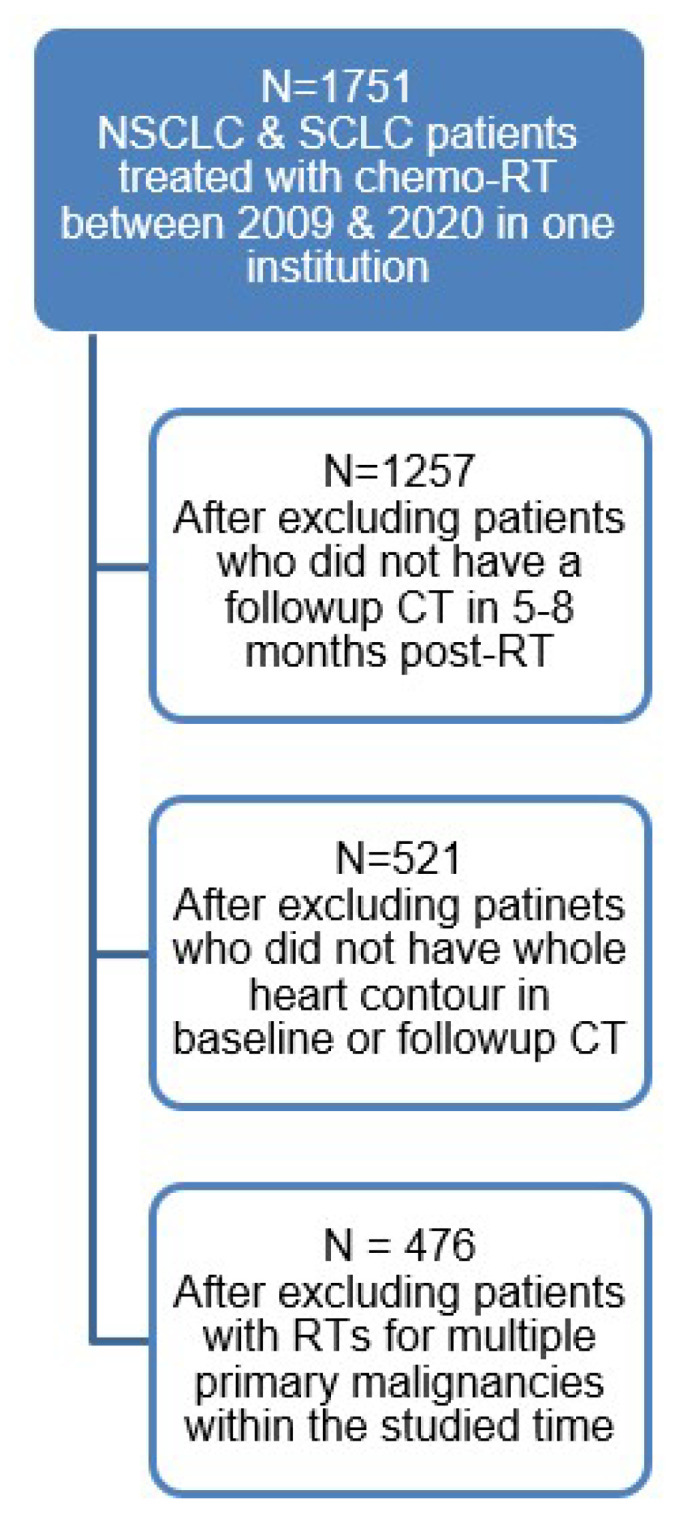
PRISMA flow diagram of patient selection.

Demographic data were retrieved from PERSIMUNE [30]. ICD10 codes for CVD were I20-I25 and I50. Cardiovascular events separated by ≤28 days were counted as only one. In such cases, only the first diagnosis entry was kept. The last follow-up date in our study was 27 February 2021. Median follow-up was 26.7 months. The total number of patients with pre- and post-RT images qualifying for our study was 476 (see Appendix A, Figure A1). Data are available in Supplementary Materials, Tables S1–S4.

### 2.2. Image Analysis

We aimed at analyzing data at the 6-month follow-up. However, to account for timing variation and some images that did not include the whole heart, we collected all follow-up images within 5–8 months post-RT. We deformably registered follow-up images on baseline images using Plastimatch software [31]. Baseline images were plan CTs. The heart and its 4 mm outmost layer (the pericardial sac) were contoured on standard-of-care (pre-RT) baseline (i.e., plan) CT scans using RootPainter [32].

**Figure 1:**
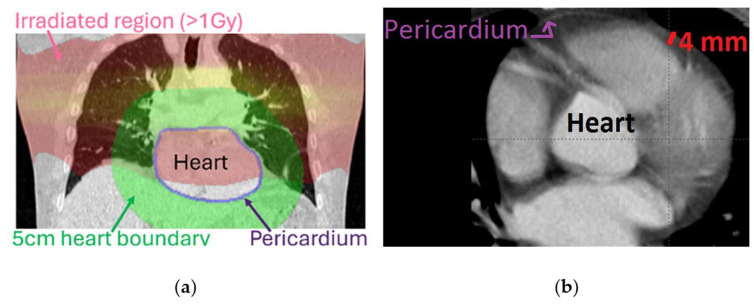
(**a**) In a patient example, pink = area where voxels received >1 Gy; green = the 5 cm ring around the heart. Hounsfield unit distribution in the region within the green area that had received ≤1 Gy was used to pick the best matching follow-up CT scan for each baseline CT scan. (**b**) An axial CT slice including the whole heart and the pericardial sac. Red line = 4 mm inner distance for one point.

A Plastimatch wrapper around an ITK registration toolkit was used and initialized by aligning heart mask centers of mass. The registration performance was optimized in stages: translation, rigid, affine, b-spline 100 mm grid, b-spline 50 mm grid, b-spline 25 mm grid, and b-spline 12 mm grid. We used a gradient-boosted tree minimizer to minimize the loss function, which included the heart contour difference (using Dice coefficient) with ground truth (heart contour generated by TotalSegmentator) [33]. Several patients had multiple follow-up images within our time-frame of interest with different scanners/reconstruction-kernels (see Appendix B, Table A1). To find the follow-up image best matching each baseline image in terms of reconstruction kernel and scanner, we calculated the average difference in voxel Hounsfield unit (HU) values between the baseline and follow-up image sets in an out-of-field region near the heart. The selected region was the part of a 5 cm ring around the heart mask that had received ≤1 Gy radiation dose (Figure 1). For each patient, the deformably registered follow-up scan with the least difference in HU distribution in this region with the baseline CT was selected. HU was binned from –1000 to 1000 in one-unit increments. Figure 1 also shows an example of how the pericardial sac is seen on a CT scan.

Thirty-nine cases had both baseline CTs and follow-up CTs without contrast enhancement, and 249 cases had both CTs with contrast enhancement. The rest had either baseline CT or follow-up CT contrast-enhanced (the majority were within the latter group). To alleviate the effect of contrast enhancement differences, we used the ComBat imaging biomarker harmonization method [34]. ComBat requires the user to separate data into batches based on the characteristic for which image features/biomarkers need to be harmonized. For the analyses that included only baseline CTs, we used two batches: contrast-enhanced and non-contrast-enhanced. For analyzing imaging biomarkers that compared pre- and post-RT images, we used four batches:

- Batch1: Both baseline and follow-up CTs without contrast enhancement;
- Batch2: Both baseline and follow-up CTs with contrast enhancement;
- Batch3: Baseline CT with and follow-up CT without contrast enhancement; and
- Batch4: Follow-up CT with and baseline CT without contrast enhancement.

Tissue composition was labeled using the HU ranges reported in Table 2 based on various previous studies [35,36,37,38,39]. Note that each group contains subgroups of tissue composition, some normal and some abnormal; thus, labels are not abnormality names. Also, the order in the table does not reflect the progress order of pericardial tissue damage (for instance, it does not mean that excess fluid necessarily develops before or after calcification is formed).

Our three image biomarkers were:

Biomarker 1. Volume associated with an HU change:

This biomarker showed the extent of HU changes for most pericardial voxels and whether there was a dose-dependency. Mean volume per HU change and per dose were calculated as:

$${\Delta HUV}_{P,\Delta HU}=100\;\times \;\{\sum_{\vartheta \in \;\Delta HU\;}V_{\vartheta ,P}\}/\{\sum_{\vartheta \;}V_{\vartheta ,P}\}$$

(1) $${\bar{\Delta HUV}}_{\Delta HU,D_{per}\;}={mean}_{P\in D_{per}\;}\{{\Delta HUV}_{P,\Delta HU}\}$$

where P,$\;\Delta HU,\;{V,\;D}_{per}\;and\;\vartheta$ represent patient, baseline minus follow-up HU ${(\Delta HU=\;HU}_{baseline}-{HU}_{followup})$, volume, mean pericardium dose, and pericardium voxel, respectively. Equation (1) calculates percent pericardial volume associated with each ΔHU averaged across patients receiving $D_{per}$ dose ($\bar{\Delta HUV}$ is ΔHUV when averaged across patients within $D_{per}$ groups).

Biomarker 2. Tissue mass change:

This biomarker calculated the voxel-based mass change (VMC) in each tissue composition HU range per dose averaged across all patients as:

$${VMC}_{P,T,D_{vox}}=100\times \{\sum_{\vartheta \in D_{vox}\;\&\;T}{\Delta HU}_{\left(\vartheta \right)}\times V_{\vartheta }\}/\{\sum_{\vartheta }\left({HU}_{baseline\left(\vartheta \right)}+1000)\times V_{\vartheta }\right\}$$

(2) $${\bar{VMC}}_{T,D_{vox}}={mean}_{P\;}\{{VMC}_{P,T,D_{vox}}\}$$

where T, P,$\;{\Delta HU}_{\left(\vartheta \right)},\;{V,\;D}_{vox}\;and\;\vartheta$ represent tissue HU range, patient, baseline minus follow-up HU ${({\Delta HU}_{\left(\vartheta \right)}=\;HU}_{baseline(\vartheta )}-{HU}_{followup(\vartheta )})$ for voxel ϑ, volume, voxel dose, and pericardium voxel, respectively. $\bar{VMC}$ is VMC when averaged across all patients.

Biomarker 3. Tissue volume change:

This biomarker calculated the volume change in each tissue composition HU range per dose as:

(3) $${\Delta V}_{P,T}=100\times \{\frac{\sum_{\vartheta \in \;T(baseline)\;}V_{\vartheta }-\;\sum_{\vartheta \in \;T(followup)\;}V_{\vartheta }\;}{\sum_{\vartheta \;}V_{\vartheta ,P}}\}$$

where T,  P , V and ϑ represent tissue HU range, patient, volume and pericardium voxel respectively.

The above image biomarkers were relative (baseline minus follow-up) values. Knowing that heart and thus pericardium have normal volume/mass changes in each person [40,41], we applied normalization: in Equations (1) and (3), volumes were divided by the total pericardium volume per patient, and, in Equation (2), masses were divided by total pericardium mass per patient.

Various code packages were used, and analyses were conducted partly in Python 3.13 and partly in MATLAB R2017a. References to the code packages used are provided when each package is mentioned. To remove the noise effect, we analyzed average biomarker values across patients and smoothed biomarker values using the smoothdata function in MATLAB (which calculates “moving average of the elements of a vector using a fixed window length that is determined heuristically” [42]).

## 3. Results

### 3.1. Registration

Registration was tested on 53 baseline and follow-up pairs. Dice coefficients for the heart contours were 0.869 ± 0.124.

### 3.2. Data Harmonization

**Figure 2:**
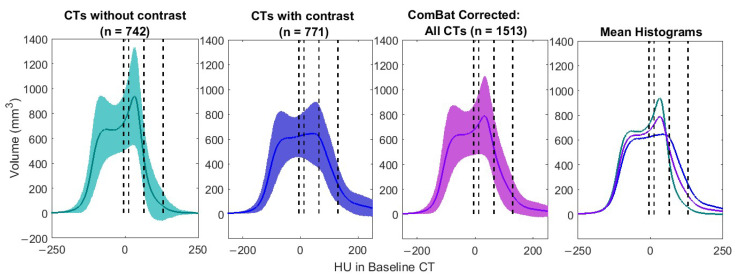
Harmonization for contrast enhancement using ComBat in 1513 baseline CT scans. Left to right: Histogram of pericardium Hounsfield units (HUs) in 742 non-contrast-enhanced CT scans (green solid line = mean; green shading = standard deviation), histogram of pericardium HUs in 771 contrast-enhanced CT scans (blue solid line = mean; blue shading = standard deviation); histogram of pericardium HUs in 1513 CT scans after correction (purple solid line = mean; purple shading = standard deviation); and mean histograms overlaid. Vertical dashed lines separate composition HU ranges based on Table 2.

**Figure 3:**
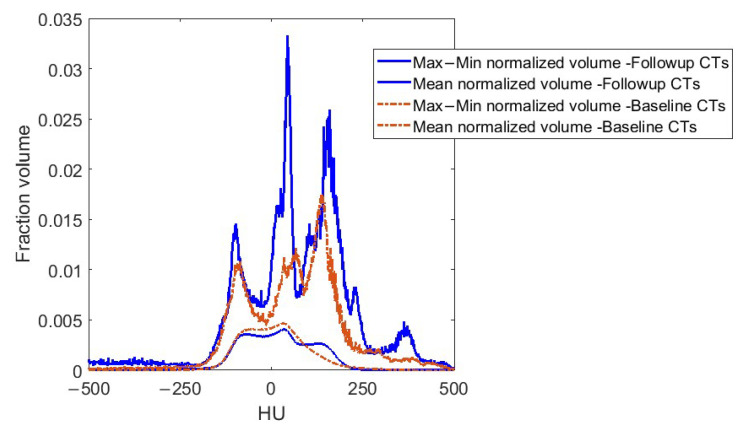
Histogram of pericardial Hounsfield units (HUs) in the 476 baseline and follow-up images in this study after application of deformable image registration and harmonization. Volumes are normalized (divided by pericardium volume), i.e., the *y* axis is a fraction volume of the pericardium. Solid lines = maximum minus minimum values across patients. Dashed lines = mean values across patients.

Figure 2 shows ComBat results for 1513 baseline images. These were the baseline images from the original 1751 patient set, excluding images in which the whole heart was not captured. Figure 3 shows ComBat results for the HU histograms (variation and mean) of both baseline and follow-up images from the main 476 patients.

### 3.3. Biomarker 1: Volume Associated to an HU Change

**Figure 4:**
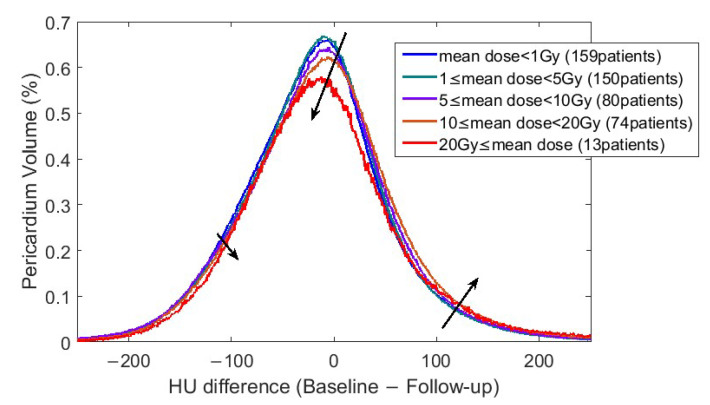
$\Delta \bar{HUV}$ grouped with respect to mean pericardium dose (EQD2). The *y* axis shows the fraction of pericardium volume. Black arrows highlight main distribution change trends with dose increase.

Figure 4 shows Equation (1) analysis and demonstrates how mean pericardium dose affected the change of HU in pericardium. Patients were grouped based on average dose to pericardium: [0,0.2) Gy, [0.2,2) Gy, [2,5) Gy, [5,12.5) Gy, [12.5,20) Gy, and ≥20 Gy. Pericardial volume associated with mean of $\Delta \bar{HUV}$ distribution was significantly correlated with mean pericardial dose (Spearman *p* value = 0.002).

### 3.4. Biomarker 2: Tissue Mass Change

**Figure 5:**
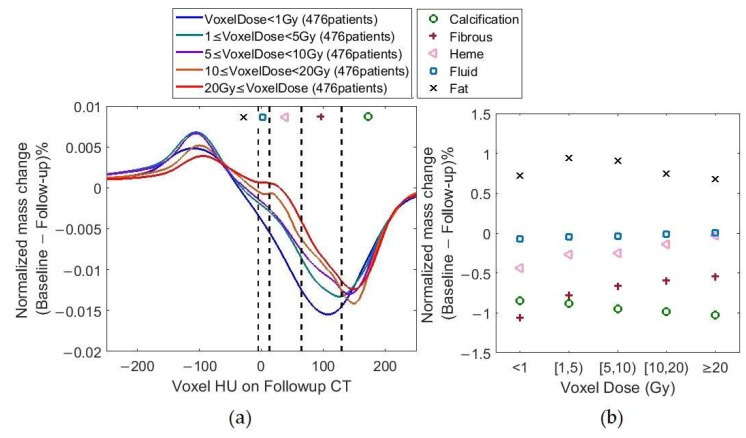
$\bar{VMC}s$ per (**a**) voxel HU and (**b**) tissue type when voxels at follow-up were used for *x* axis. Doses are EQD2 values.

Figure 5 shows $\bar{VMC}$ (Equation (2)) analysis. In Figure 5b, composition-based values were calculated using the HU ranges in Table 2. All normalized mass changes in HU range associated with one tissue composition (e.g., Calcification) in Figure 5a were summed up to generate Figure 5b. Dose correlation *p* values were <0.05 for Fibrous, Heme, and Fluid HU ranges.

### 3.5. Biomarker 3: Tissue Volume Change

**Figure 6:**
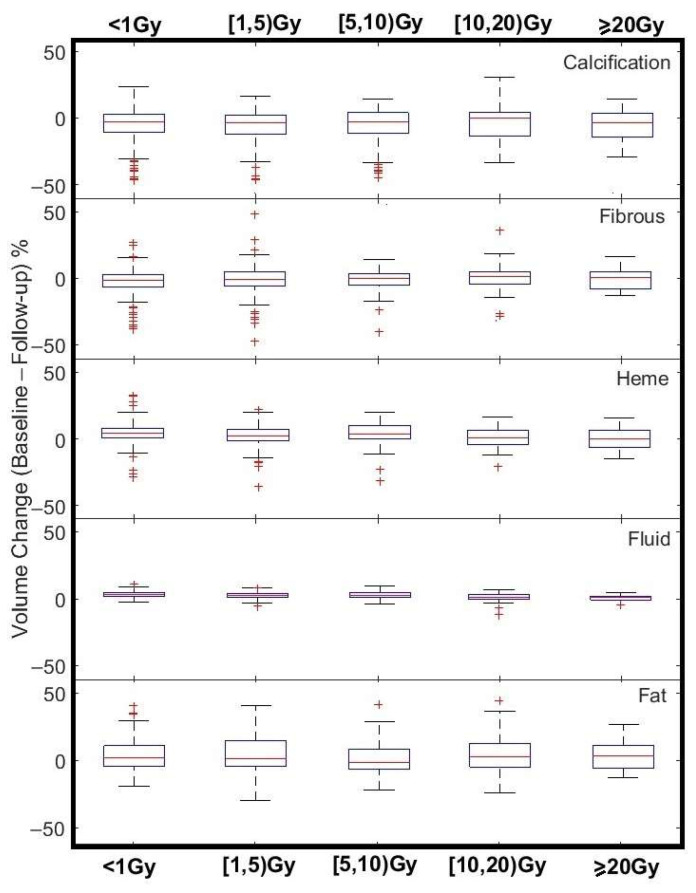
Box plot demonstration of ΔV variation for different tissue types (rows) and in various pericardial mean dose (EQD2) bins. Tops and bottoms of each box plot are the 25th and 75th percentiles, respectively. The line in the middle of each box is the median.

Figure 6 provides a visualized summary of Equation (3) results for various dose groups. Volume change in Fibrous, Heme, and Fluid HU ranges were significantly correlated with mean pericardial dose (Spearman *p* values ≤ 0.03).

### 3.6. Survival Analysis

**Figure 7:**
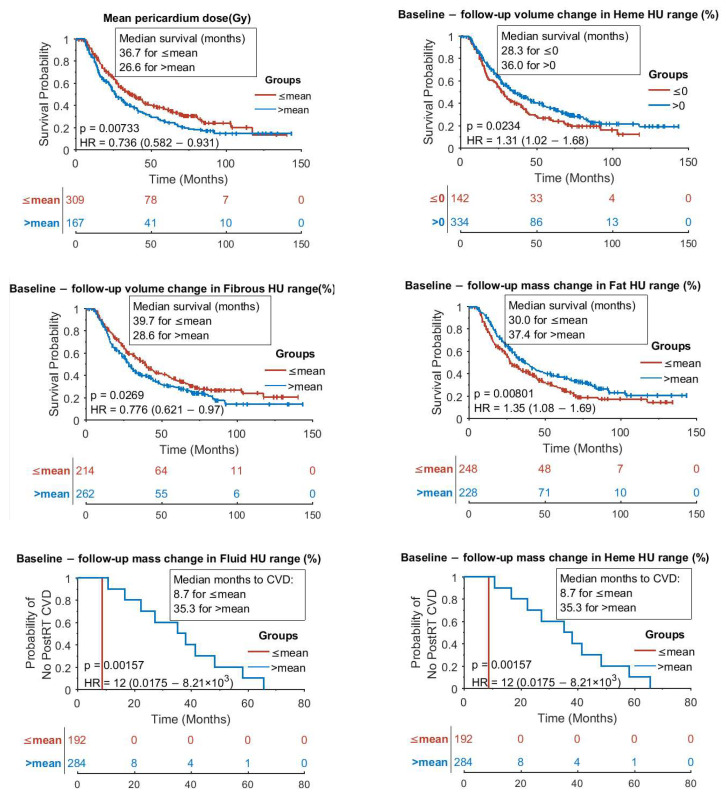
Pericardium dose response in Kaplan–Meier analysis: the 6 panels show significant difference in survival post-RT for patients with respect to mean EQD2, volume changes in Heme and Fibrous HU ranges, and mass change in Fat HU range. Also, there is a significant difference in time to the first post-RT CVD diagnosis for patients with respect to mass change in Heme and Fibrous HU ranges. Mean value for each curve is the mean value of covariate among the 476 patients. Log rank *p* values, hazard ratios (HR), and HR 95% confidence intervals (lower-upper) are shown on the panels.

**Figure A2:**
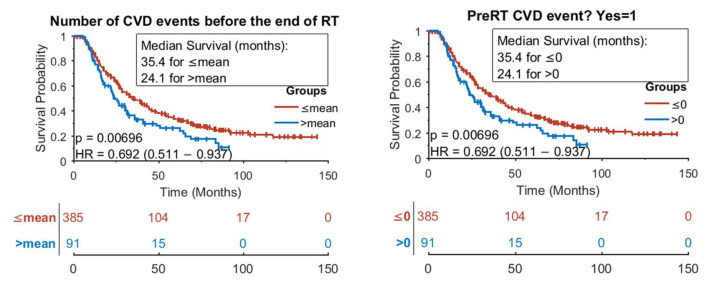
Kaplan–Meier curves show significant difference in survival post-RT for patients with respect to experiencing pre-RT CVD events.

**Figure A3:**
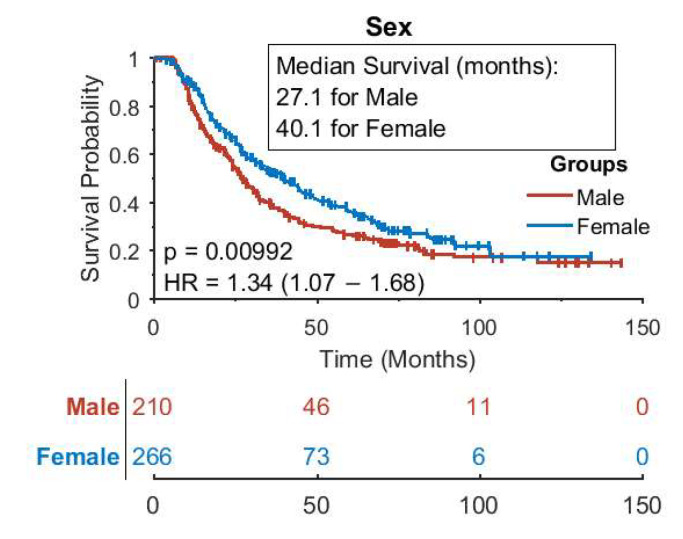
Kaplan–Meier curves show significant difference in survival post-RT for patients with respect to their sex.

**Figure A4:**
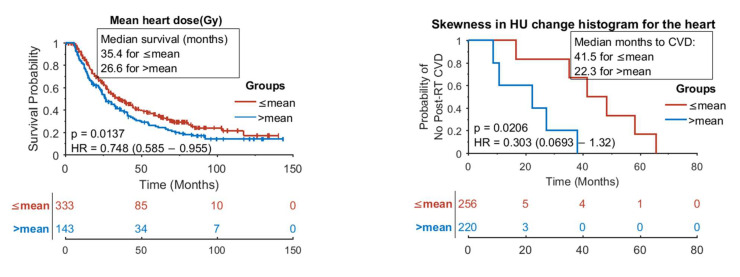
Whole heart-related Kaplan–Meier results: Kaplan–Meier curves show significant difference in survival post-RT for patients with respect to mean heart dose and significant difference in time to the first post-RT CVD diagnosis for patients with respect to ΔHU  histogram skewness.

**Figure A5:**
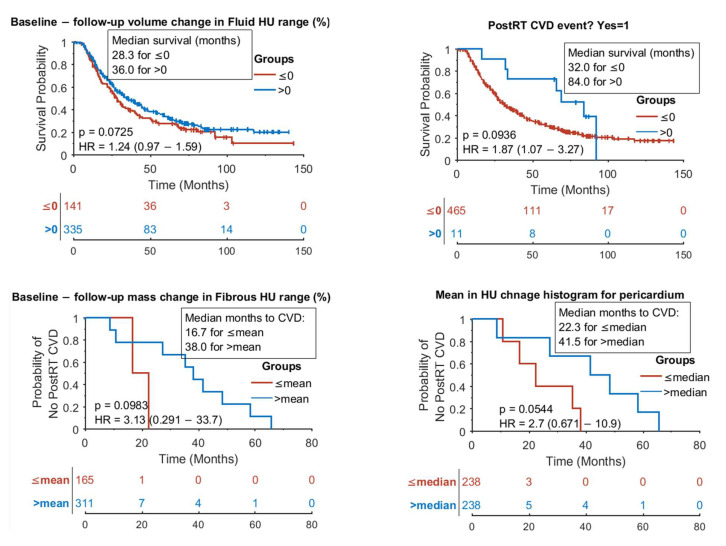
Covariates with marginal significance in survival or post-RT CVD.

Figure 7 summarizes the Kaplan–Meier analysis results for pericardium dose response. The curves were produced using a MATLAB package [43]. The list of the parameters and endpoints studied are provided in Appendix C. Life expectancy in our studied lung cancer group was adversely affected by pre-RT CVD diagnoses (Figure A2). Also, Figure A3 shows that males had shorter life expectancy, and Figure A4 shows the parameters related to the whole heart that had significant effects on survival and post-RT CVD diagnoses. Finally, Figure A5 shows the covariates with marginal significance.

## 4. Discussion

Although the correlation between pericardium sac health and overall cardiac health has been studied in the cardiology literature [14,29,44,45,46,47], in radiation oncology this correlation has not received its deserved attention. This study shows that the pericardium demonstrates RT-dose-dependent composition changes at 6 months post-RT, suggesting that assessing these changes could guide the cardio-oncology team in better managing lung cancer patients.

The histogram of HU change in the pericardium from baseline to follow-up had significant correlation with mean dose to pericardium, patient age, and patient sex. It was also correlated with the number of CVD diagnoses pre- and post-RT.

The dose-based curves in Figure 5a show a shift to the right with a dose increase in the *y* < 0 half of the figure, meaning that as dose increased, voxels with mass increase from baseline to follow-up shifted from the Fluid HU range (−5 to 12) to belonging to Calcification HU range ≥130. This trend seemed to stop for voxels with HUs >250. This is in line with findings that showed the importance of constrictive pericarditis and rarity of calcified constrictive pericarditis [45,48,49]. In Figure 5b, the observed voxel-based mass change from baseline to follow-up was inversely related to dose (Pearson correlation factors ≥ 0.96 and *p* values ≤ 0.03) for Fibrous, Heme, and Fluid HU regions despite considering only five data points (for the five-dose bins). The same phenomenon was observed when assessing images from the 39 patients whose baseline and follow-up CTs were both non-contrast-enhanced (figure not included due to similarity to Figure 5). Note that the pericardium is often thinner than 4 mm, and our analysis included some myocardial tissue (Figure 1b) with HU values within Fibrous HU range. A decrease in mass for Fibrous with an increase in RT dose could be due to more pericardial thickening and less myocardial tissue being present in the 4 mm studied region.

Kaplan–Meier curves in Figure 7 show that longer patient survival was significantly correlated with pericardial mass decrease in Fat, volume decrease in Heme, and volume increase in Fibrous HU ranges, whereas longer time to a post-RT CVD diagnosis was highly correlated with pericardial mass decrease in Fluid and Heme HU ranges. These findings agree well with studies that show poor prognoses in lung cancer patients with constrictive (fibrous and thickened) pericarditis and pericardial effusion (with various fluid compositions including blood) [17,22,36,47,48,49,50]. It should be noted that fat-related results could be confounded by overall weight change in cancer patients. Again, note that the 4 mm contour included some myocardial tissue with HU values within Fibrous HU range. This explains why the larger volume in the Fibrous HU range was associated with longer survival: the larger fibrous volume could be a result of a thinner pericardium with more myocardial tissue detected within the Fibrous range. Furthermore, patient sex, mean heart dose, cardiac diagnoses prior to RT, and mean pericardium dose were highly correlated with patient survival (Appendix D and Figure 7). The probability of post-RT CVD diagnoses was also highly correlated with the histogram of HU change in the whole heart (Appendix D), suggesting that a similar analysis in cardiac substructures other than the pericardium could result in clinically relevant findings.

Our studied cohort included 413 NSCLC patients and 63 SCLC patients. We repeated the analyses in the NSCLC subgroup, and results were similar to the entire patient cohort as reported here (see Appendix E, Table A2 and Table A3). However, we did not perform the analysis in the SCLC cohort because the sample size was insufficient.

We acknowledge specific limitations in our study:

Limitation 1: Variation in HU values generated by different scanners of different manufacturers using various reconstruction kernels (Appendix B) added complexity to our retrospective analysis of standard-of-care images. Although our results look promising, they should be verified in an external series with other scanners and reconstruction kernels. Our chosen image registration and harmonization tools (while widely used and having gained approval in this field) added potential uncertainties to our results. For example, the pericardium mass in follow-up CTs was on average 1.46% (~1 g) larger than that in baseline CTs in our patient cohort. This meant that patients overall had an increase in their pericardial mass irrespective of the dose. However, this mass increase could be an image registration error. If the increase was definitively proven to be an artifact and removed, there would be a baseline change in Figure 5b, meaning that the starting point for the five composition-based mass change curves in Figure 5b would be zero. A future study with a longer follow-up period could shed light on whether this mass change is an artifact, a transient pericardial response to radiation, or something else.

Limitation 2: Our HU range grouping as shown in Table 2 should be read with caution. For example, pericardial effusion is one of the most common post-RT cardiotoxicity diagnoses (agreeing with our finding that lower masses in Fluid and Heme HU ranges were associated with longer time to a post-RT CVD diagnosis in Figure 7); however, effusion fluid composition and HU values vary and can include portions of both Fluid and Heme HU ranges in Table 2 [51,52]. That could explain why patients in the mass change group for Fluid and Heme in the analysis of time to post-RT CVD (Figure 7’s two final panels) were the same, producing the same Kaplan–Meier results. A finer HU binning with more tissue composition groups could help with more interpretable results; however, the effect of uncertainties (overlapping HU ranges) should be well managed (e.g., there is a large overlap between exudative pericardial effusion HUs and transudative pericardial effusion HUs: 14.85 ± 10.7 HU vs. 1.13 ± 4.3 HU [36]).

Limitation 3: Our primary endpoints were death and post-RT CVD diagnoses. However, the number of events in the latter group was quite low. Only 11 of the 476 patients had a diagnosis for post-RT CVD. The Kaplan–Meier curves in Figure 7’s two final panels were clearly affected since there were no CVD events in one of the analyzed groups. External validation in cohorts with more CVD events could be valuable. This is a limitation in any lung cancer population study because of relatively short survival as well as the intertwined connection between cardiac and pulmonary systems, making it difficult to separate CVD symptoms from those of lung cancer [53,54,55]. Wang et al. studied 278,418 lung cancer patients diagnosed from 1990 to 2020 [56]. They found 12,584 diagnoses of CVD within a median follow-up of 9 months (interquartile range 3–27 months). They concluded by emphasizing “the necessity of integrating cardiovascular risk assessment and management into lung cancer treatment protocols, particularly during the first month after diagnosis and for younger or high-risk subgroups.” The fact that several retrospective studies have found significant correlation between radiation dose to the heart (or its substructures) and survival in lung cancer patients in the absence of clinical reports of CVD diagnoses for the majority of these patients may well point to a silent cardiac health decline (signifying the need for markers) [53].

Limitation 4: Although we analyzed volume and mass change between baseline and follow-up, we did not assess whether associated voxels were within clusters (connected voxels). Similarly, we did not separate parietal and visceral voxels in the pericardium, nor did we separate epicardial fat from other fatty tissue within the pericardial sac. We will consider performing such analyses in future work.

Limitation 5: Although pericardial abnormalities can be identified on CT scans, automated pericardium contouring on CT scans has not been well investigated. Manual contouring is cumbersome, especially in non-contrast-enhanced CT scans. In RT, less attention has been given to this cardiac substructure, and therefore its accurate contouring has not been a focus of interest. Most pericardium-related studies in RT are in fact reporting on their analysis of the whole heart including the pericardial sac and not the sac itself [20,21]. Our work and recently raised attention to pericardial analysis [24,26] may increase interest in auto-contouring tools for the pericardial sac. Several other cardiac substructures are already being auto-segmented [32,33].

Limitation 6: The morphological presentations of RT-induced pericardial disease are mostly in fibrinous (inflammation with thickening), effusion (fluid containing protein/blood), and abnormal fibrous (fibrosis, constrictive, or calcified tissue) groups [57]. A future longitudinal study of changes at more than one post-RT timepoint (e.g., also at 12-month) could potentially help to clarify the pathophysiological mechanisms of RT-induced pericardial damage. Such longer follow-up time will also allow manifestation of detectable calcification, which is an important cause of cardiac death.

Limitation 7: Although our study reports on a potentially important finding for identifying patients in need of cardio-oncologic care in a timely manner post-RT, it will benefit from including the effect of tumor stage and histology in future research. As an example, squamous cell carcinoma is generally associated with poor prognosis and is more prevalent in men. Such histological distribution difference may partly explain the observed sex-based survival disparity. Another example is that it is generally known that patients with peripheral T1 or T2 tumors treated with SBRT have better survival than patients with locally advanced tumors with lymph node involvement near the heart.

## 5. Conclusions

As Logotheti et al. noted: “Cardiovascular diseases (CVD) represent a clinically important, but mechanistically understudied complication, which interfere with the continuation of best-possible care, induce life-threatening risks, and/or lead to long-term morbidity” [6]. Our study showed that pericardium composition distribution had dose-dependent changes as early as 6 months after RT. These changes are detectable on the standard-of-care CTs and can potentially serve as screening measures and early markers of life-compromising cardiotoxicity.

## Figures and Tables

**Table 1:** Patient characteristics; n is the number of patients (out of 476) within each group.

Characteristic	n	Characteristic	n	Characteristic	n
Sex		Mean heart dose		Patients with	
Female	266	>10 Gy	68	History of RT for other primary cancers ^3^	0
Male	210	<1 Gy	188	History of previous primary cancers	178
Age at treatment ^1^		Max heart dose ^2^		Pre-RT cardiovascular diseases	91
≥65	381	>15 Gy	292	Post-RT cardiovascular diseases	11
Diagnosis		Mean pericardium dose		Survival ≤2 years post-RT	171
SCLC	63	>10 Gy	87	Survival >5 years post-RT	28
NSCLC	413	<1 Gy	159	Image contrast enhancement	
RT fractions				Contrast enhanced baseline CTs	260
≤8	183			Contrast enhanced follow-up CTs	426

^1^ Age: 28–93 (median 69) years. ^2^ Median of mean heart doses was 1.9 Gy (max, 32.1 Gy). ^3^ Within the studied period of time

**Table 2:** Hounsfield unit (HU) range associated with different tissue compositions. For brevity, the composition label reported here is used for each HU range throughout the paper.

Tissue Composition/s	HU Range	Label
Calcification, calcified constrictive tissue, malignancy	HU ≥ 130	Calcification
Fibrosis, constrictive tissue, adjacent myocardial tissue	129 ≥ HU ≥ 65	Fibrous
Normal pericardium, thickened pericardium, hemopericardium	64 ≥ HU ≥ 13	Heme
Effusion, normal fluid	12 ≥ HU ≥ −5	Fluid
Fat (including normal fatty tissue)	HU ≤ −6	Fat

## Data Availability

Anonymized data (after deformable image registration and before the application of ComBat) are included here as Supplementary Materials.

## Supplementary Materials

The following supporting information can be downloaded at: https://www.mdpi.com/article/10.3390/cancers17162635/s1, Table S1: Demographic and health history data, Table S2: Voxel-level data on total mass per HU bin, Table S3: Voxel-level data on mass difference between baseline and follow-up per HU bin, Table S4: Volume difference between baseline and follow-up per HU change.

## Abbreviations

The following abbreviations are used in this manuscript:

CT	Computed tomography
CVD	Cardiovascular diseases
EQD2	Equivalent Dose in 2 Gy fractions
fx	fraction
HU	Hounsfield unit
ICD10	International Classification of Diseases, 10th Revision
PRISMA	Preferred Reporting Items for Systematic Reviews and Meta-Analyses
RT	Radiotherapy

## Appendix B

**Table A1:** Manufacturer-based variation in CT machines and reconstruction kernels.

Manufacturer	Machine—Reconstruction Kernel/Method
GE Medical Systems	Revolution Apex—Standard
	Revolution CT—Standard
	Discovery LS—Standard
	Revolution CT—STND#
	Discovery MI—Standard
	Discovery 710—Lung
	Discovery 710—Standard
Philips	GEMINI TF TOF 64—C
	GEMINI TF TOF 64—B
	GEMINI TF TOF 64—D
	GEMINI TF TOF 64T—B
	GEMINI TF TOF 16—B
	Brilliance 16P—C
	Brilliance 64—L
	Brilliance 64—C
	Brilliance 64—D
	Brilliance 64—B
	Brilliance 64—UB
	Brilliance 64—YA
	Precedence 16p—B
	iCT 256—B
	iCT 256—C
	Mx8000 IDT 16—C
	Iqon—Spectral CT—C
Toshiba	Aquilion—FC11
	Aquilion—FC12
	Aquilion—FC50
	Aquilion—FC51
	Aquilion PRIME—FC56
	Aquilion PRIME—FC0B
	Aquilion ONE—FC0B
	Aquilion ONE—FC51
	Aquilion ONE—FC56
Siemens	Biograph 40—B40f
	Biograph 40—B30f
	Biograph 40—B19f
	Biograph 40—B20f
	Biograph 64—B40f
	Biograph 64—[‘130f’,’3’]
	Biograph 64—B30f
	Biograph 64—B19f
	Biograph 64—B70f
	Sensation 16—B40f
	Sensation 64—B30f
	SOMATOM Definition—B60f
	SOMATOM Definition Flash—B50f
	SOMATOM Definition Flash—[‘j70h’,’3’]
	SOMATOM Definition Flash—[‘170f’,’3’]
	SOMATOM Definition Flash—[‘130f’,’2’]
	SOMATOM Definition Edge—[‘170f’,’3’]
	SOMATOM Definition Edge—B40f
	SOMATOM Force—[‘Bv40d’,’3’]
	SOMATOM Force—[‘Qr40d’,’3’]
	SOMATOM Definition AS—B30f

## Appendix C

**The list of variables studied:**

**Demographic:**

‘Sex f=1’

‘Age at treatment’

‘Diagnosis NSCLC=1 SCLC=0’

* * 

**Health history:**

‘PreRT CVD event? Yes=1’

‘Number of CVD events before the end of RT’

‘Other cancers before this? Yes=1’

‘Other cancers after this? Yes=1’

* * 

**Dosimetric:**

‘Mean heart EQD2 (Gy)’

‘Max heart EQD2 (Gy)’

‘Mean pericardium EQD2 (Gy)’

‘Max pericardium EQD2 (Gy)’

‘Number of fractions’

‘Dose per fraction (Gy)’

* * 

$\Delta \bar{HUV}$ **(Equation (1))**

‘Plan-follow-up volume for HU range: calcification volume%’

‘Plan-follow-up volume for HU range: Fibrous volume%’

‘Plan-follow-up volume for HU range: Heme volume%’

‘Plan-follow-up volume for HU range: Fluid volume%’

‘Plan-follow-up volume for HU range: Fat volume%’

‘Distribution mean-mode (skewness) pericardium’

‘Mean (DeltaHU) for pericardium’

‘Volume @ Mean (%) for pericardium’

‘Distribution mean-mode (skewness) heart’

‘Mean (DeltaHU) for heart’

‘Volume @ Mean (%) for heart’

* * 

$\bar{VCM}$ **(Equation (2))**

‘Mass Change in pericardium for Calcification (%) baseline CT bin’

‘Mass Change in pericardium for Fibrous (%) baseline CT bin’

‘Mass Change in pericardium for Heme (%) baseline CT bin’

‘Mass Change in pericardium for Fluid (%) baseline CT bin’

‘Mass Change in pericardium for Fat (%) baseline CT bin’

‘Mass Change in pericardium for Calcification (%) follow-up CT bin’

‘Mass Change in pericardium for Fibrous (%) follow-up CT bin’

‘Mass Change in pericardium for Heme (%) follow-up CT bin’

‘Mass Change in pericardium for Fluid (%) follow-up CT bin’

‘Mass Change in pericardium for Fat (%) follow-up CT bin’

* * 

**Endpoints:**

‘Dead within 2 years? Yes = 1’

‘PostRT CVD event? Yes = 1’

‘Months from the end of RT to the 1st CVD event’

‘Number of CVD events after the end of RT’

‘Dead? Yes = 1’

‘Months of survival after the end of RT’

## Appendix D

Additional Kaplan–Meier analysis

## Appendix E

Kaplan–Meier analysis in the 413 NSCLC patients

**Table A2:** Survival.

Covariate	*p* Value	HR (95% Confidence Interval)	Median Survival Months
Mean pericardium dose	0.0207	0.751 (0.583–0.969)	(≤mean) = 36.7 (>mean) = 26.6
Mean heart dose	0.0206	0.745 (0.571–0.971)	(≤mean) = 35.4 (>mean) = 26.7
Baseline—follow-up mass change in Fat HU range (%)	0.023	1.32 (1.04–1.67)	(≤mean) = 28.0 (>mean) = 36.7
Baseline—follow-up volume change in Fluid HU range (%)	0.0424	1.3 (0.994–1.7)	(≤0) = 28.3 (>0) = 36.0
Baseline—follow-up volume change in Heme HU range (%)	0.0101	1.38 (1.06–1.8)	(≤0) = 26.9 (>0) = 36.7
Baseline—follow-up volume change in Fibrous HU range (%)	0.0242	0.76 (0.598–0.965)	(≤mean) = 39.7 (>mean) = 28.6
Pre-RT CVD events (yes = 1)	0.0056	0.673 (0.488–0.926)	(≤0) = 36.7 (>0) = 24.1
Number of CVD events before the end of RT	0.0056	0.673 (0.488–0.926)	(≤0) = 36.7 (>0) = 24.1
Patient sex (female = 1)	0.00472	1.41 (1.1–1.8)	(≤0) = 26.7333 (>0) = 41.4667
Post-RT CVD events (yes = 1)	0.168	1.68 (0.932–3.03)	(≤0) = 31.4 (>0) = 69.1
Number of CVD events after the end of RT	0.168	1.68 (0.932–3.03)	(≤0) = 31.4 (>0) = 69.1

**Table A3:** Time to post-RT CVD.

Covariate	*p* Value	HR (95% Confidence Interval)	Median Months to Post-RT CVD
Baseline—follow-up mass change in Fluid HU range (%)	0.0027	11 (0.0217–5.58 × 10^3^)	(≤mean) = 8.7 (>mean) = 38.0
Baseline—follow-up mass change in Heme HU range (%)	0.0027	11 (0.0217–5.58 × 10^3^)	(≤mean) = 8.7 (>mean) = 38.0
Baseline—follow-up mass change in Fibrous HU range (%)	0.139	2.82 (0.292–27.1)	(≤mean) = 16.7 (>mean) = 38.0
Skewness in HU-change histogram for the heart	0.0197	0.296 (0.0675–1.29)	(≤mean) = 48.4 (>mean) = 22.3
Mean in HU-change histogram for pericardium	0.0544	2.89 (0.591–14.2)	(≤median) = 16.7 (>median) = 41.5
